# Feasibility of quantification based on novel evaluation with stool DNA and fecal immunochemical test for colorectal cancer detection

**DOI:** 10.1186/s12876-022-02470-z

**Published:** 2022-08-13

**Authors:** Hongli Xu, Huixin Chen, Junjie Hu, Zhiguo Xiong, Dongqing Li, Shun Wang, Jing Yu

**Affiliations:** 1 Department of Abdominal Medicine, Colorectal Cancer Clinical Research Center of Hubei Province, Hubei Cancer Hospital, Tongji Medical College, Huazhong University of Science and Technology, Wuhan, 430079 China; 2 Department of Blood Transfusion, Wuhan Hospital of Traditional Chinese and Western Medicine, Tongji Medical College, Huazhong University of Science and Technology, Wuhan, 430022 China; 3 Department of Gastrointestinal Surgery, Hubei Cancer Hospital, Tongji Medical College, Huazhong University of Science and Technology, Wuhan, 430079 China; 4 Department of Microbiology, School of Basic Medical of Science, Wuhan University, Wuhan, 430071 China

**Keywords:** Colorectal cancer, Novel evaluation, Stool DNA tests, Fecal immunochemical test, Cancer screening

## Abstract

**Background:**

Stool DNA (sDNA) tests and fecal immunochemical test (FIT) are used for the detection of colorectal cancer (CRC). Here we performed a novel evaluation using sDNA and FIT to assess their performance in CRC screening and monitoring in Hubei, China.

**Methods:**

Stool samples were collected from a high-risk population in Hubei, China (n = 359). sDNA tests and FIT were performed to test for KRAS mutations, NDRG4 and BMP3 methylation, and check hemoglobin levels. The methylation in BMP3 and NDRG4 genes was detected by TaqMan PCR method from human fecal samples. KRAS gene mutation in human fecal DNA was tested using TaqMan probe and amplification-refractory mutation system method. The colloid gold method was used for detection of hemoglobin in fecal samples. Finally, a novel evaluation by software was used to calculate the comprehensive value of the combined results for CRC detection and monitoring.

**Results:**

The sensitivity and specificity of the novel evaluation for early CRC (stage I and II), advanced adenoma (AA), and non-colon cancer neoplasm (NA) detection were 95.45% and 81.6%, 29.63% and 75.9%, and 23.08% and 75.17%, respectively. The receiver operating characteristic (ROC) curves of the combined value for the above diseases were 0.945 ± 0.015, 0.543 ± 0.055, and 0.547 ± 0.038, respectively. The levels of the novel evaluation were not significantly associated with the pathology and stage (*P* > 0.05). In 20 out of 22 CRC patients, the novel evaluation of sDNA and FIT had decreased below threshold (< 165) at after surgery.

**Discussion:**

The novel evaluation with sDNA test and FIT has increased sensitivity for screening of CRC and AA. The novel evaluation may have potential importance as an indicator of early CRC. Additionally, the dynamic changes of the comprehensive value after surgery were correlated with CRC treatment.

## Highlights

- We constructed a novel evaluation with stool DNA and fecal immunochemical test (FIT) for early colorectal cancer (CRC) detection and treatment of CRC.
- The sensitivity and specificity of the comprehensive value for early CRC diagnose were 95.45% and 81.6%, and the ROC curve was 0.945 ± 0.015.
- The novel evaluation of sDNA and FIT has decreased below threshold (< 165) after surgery. The dynamic changes of the comprehensive value were correlated with CRC treatment.

## Background

According to the 2020 statistics, colorectal cancer (CRC) ranked third among all malignant cancers in terms of incidence rates and ranked fourth as the main reason for cancer-related deaths1 in Hubei province, China [1–4]. Although the 5-year overall survival (OS) of CRC can be as high as 60% in China, patients with advanced CRC are often treated with surgery, and postoperatively, an ostomy is required [5–8]. A fistula may seriously affect the patients’ life quality [9, 10]. As most patients are diagnosed with advanced disease, diagnostic methods with increased sensitivity and specificity are urgently needed in clinical practice [11].

According to the World Health Organization, CRC is considered one of the most suitable cancers for early screening and prevention [9]. The screening of CRC has been included in the public health program in North America and Europe [3, 8, 12, 13]. Owing to benefiting from CRC screening, the incidence and mortality of CRC in the United States has continuously decreased in the past 10 years [8, 14]. Studies have reported that KRAS mutation, NDRG4 and BMP3 methylation, and immunoassay fecal immunochemical test (FIT) can be used as important biomarkers for early detection of CRC and precancerous lesions [15–17]. Here, we analyze the feasibility of a novel evaluation method with stool DNA (sDNA) test and FIT for the detection of CRC in the Hubei province of China.

## Materials and methods

In this study, we enrolled both retrospective and prospective cases to evaluated the clinical performance of this novel evaluation with sDNA test and FIT for detection of CRC. The study cohort (N = 359) included CRC patients, the screening population, and other cancer patients who were enrolled at Hubei Cancer Hospital, China, between May 2018 and November 2019. The screening population has one of the following characteristics: Positive FOBT history; Family history of colorectal cancer; Chronic diarrhea; Chronic constipation; Mucinous blood stool; Chronic appendicitis; History of mental stimulation; History of chronic biliary tract disease. Only patients for whom CRC was confirmed based on pathological evidence according to the World Health Organization criteria [7], and patients who had not undergone prior anticancer treatment were included. Further, based on TNM classification, the tumors were staged as I, II, III, or IV. The study was carried out in accordance with ethical guidelines and approved by the Ethics committee of Hubei Cancer Hospital (ethical approval number: 2018[31]). Written informed consent was obtained from individual.

Gene and methylation detection: KRAS gene mutation in human fecal DNA were detected by using TaqMan probe and amplification-refractory mutation system (ARMS) PCR method with Specific primers. The TaqMan probe was used to detect the amplification products. In combination with the highly specific heat-activated Taq enzyme and the PCR reaction program, the mutation with the lowest base difference could be specifically distinguished, and seven common mutation types on the second exon of the human KRAS gene could be detected. In addition, the reference gene ACTB was detected simultaneously by multiplex PCR to assess whether the quality of human DNA in feces samples was normal.

The CpG island cytosine methylation in the BMP3 and NDRG4 genes of human fecal samples was detected by TaqMan PCR method. The DNA was sulfite treated before detection.

**Fig. 1:**
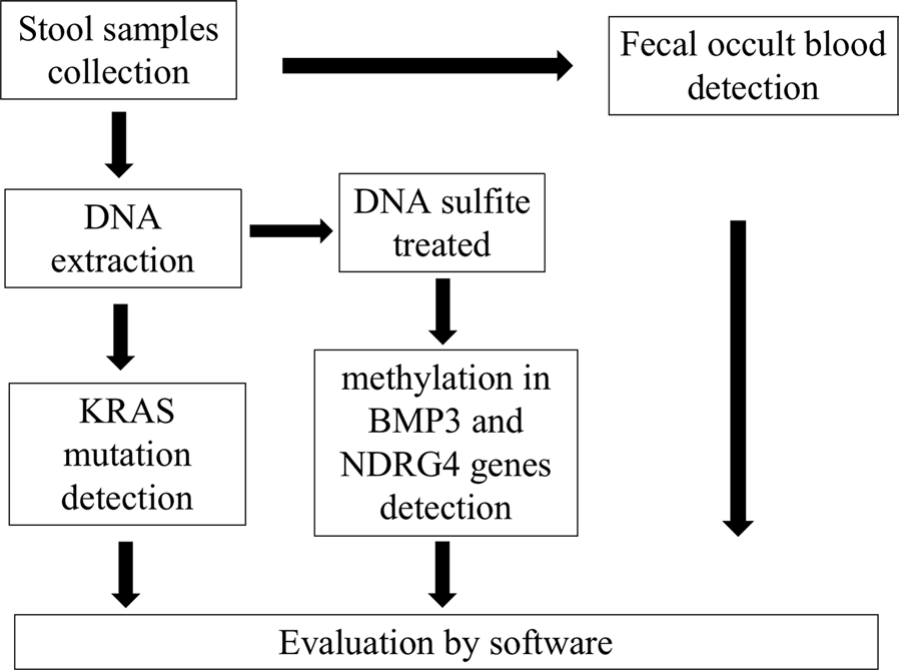
The program of sDNA tests and FIT detection and evaluation

*Hemoglobin detection*: The fecal occult blood (FOB) detection reagent (colloid gold method) used a double antibody sandwich method, containing the anti-human hemoglobin antibody prefixed to the on-membrane detection region (T) and the sheep anti-mouse polyclonal antibody in the quality control region (C). If the sample contains human hemoglobin, it will first form an antigen–antibody complex with the colloidal gold-labeled anti-human hemoglobin antibody in the colloidal gold pad, which will then be captured by the anti-human hemoglobin antibody fixed in the detection region (T) when it passes through the test area (T), and a purple-red band will appear in the test area (T) and be determined as positive. If the sample does not contain human hemoglobin, the double-antibody sandwich complex will not form in the test area (T), and therefore no purplish red bands will be formed in the test area (T), and the test area will be considered negative. Regardless of whether human hemoglobin is present in the sample, a colloidal gold-labeled mouse IgG-sheep anti-mouse polyclonal antibody complex is formed in the quality control area (C), resulting in a purplish red band. The purplish red band in the quality control area (C) is the standard to determine whether the chromatographic process is normal, and serves as the internal control standard of the reagent. The detection program was carried out as shown in Fig. 1.

The combination of Kras mutation and BMP and NDRG4 methylation was chosen as sDNA the same as a commercial kit ColoClear®, New Horizon Health Technology, China. The study was designed by the authors and funded by New Horizon Health Technology. The study followed the requirements of Technical Guidelines for Clinical Trials of In Vitro Diagnostic product from National Medical Products Administration (NMPA, Reg No. CSZ2000050), and the registration number at Clinical trials was NCT04287335. Our study with screening for CRC was one sites in Hubei Province of the whole Clinical trials (NCT04287335). A fecal immunochemical test (FIT) was used as a first-line FOBT (Wuhan Kangzhu Biotechnology Co., LTD) comparison with a commercial kit ColoClear®.

All colonoscopies were performed according to the standard quality indicators defined by the Society of Gastrointestinal Endoscopy (Olympus, CF-H170I, Japan). The most important quality indicators were qualifications and colonoscopy experience of the endoscopist, adequate bowel preparation (Ottawa bowel preparation score: 11), colonoscope withdrawal time (> 6 min), and completeness of the colonoscopy. All colonoscopies were performed by endoscopists with an experience of more than 1000 colonoscopies, the median Ottawa bowel preparation score was 5 (interquartile range: 3–8), median net withdrawal time was 10 min (interquartile range: 8–15 min), and the cecal intubation rate was 99%. The levels of CEA were detected using a chemiluminescence immunoassay (Abbott Laboratories, Chicago, USA) and their associated reagents.

### Statistics analysis

According to the results of KRAS mutation, methylation of BMP3 and NDRG4, and FOBT, a special software was used to analyze the combined detection for CRC screening with a calculated P value. The basic form of the calculation formula is where K = S + A × (FOBT positive or negative) + B × (KRAS Ct) + C × (BMP3 Ct) + D × (NDRG4 Ct). S, is a constant, and A, B, C, and D are the coefficients used in the comprehensive calculation of the four individual test results. The coefficient determination and formula shaping have been analyzed on the sample data of the training set and the validation set. The purpose is to obtain the best coefficient when the related loss function (loss function) is minimized through machine learning.

The Score calculated by formulas for the four test results is the probability that each test sample comes from a normal (negative) colorectal cancer patient and an advanced adenoma patient (positive). The positive judgment value of this detection method is 165, that is, when the Score value is less than 165, the test result of the sample is negative, and when the Score value ≥ 165, the test result of the sample is positive. And colonoscopy or other clinical procedures were recommended for further diagnosis. The specificity and sensitivity of this evaluation was analyzed by ROC curves. Statistical analysis was performed with SPSS version 20.0 (IBM Corp., Armonk, NY, USA). The *t*-test and chi-square test were used to analyze the differences between different groups with different results. *P* < 0.05 was considered to indicate statistical significance.

## Results

### Study cohort

**Fig. 2:**
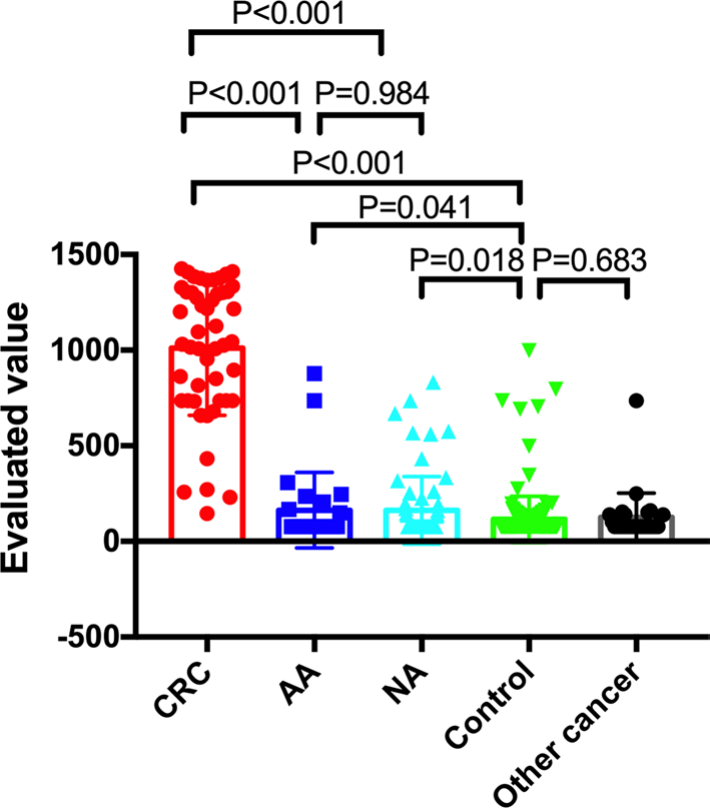
The evaluation value of CRC patients and screening of subjects. The evaluation value of CRC patients was significantly higher than those of AA, NA, healthy controls, and other cancer patients. The evaluation value of AA and NA patients were significantly higher than those in healthy controls. No significant differences were seen between AA and NA patients, other cancer patients, and healthy controls

This study included 14 patients with CRC and 345 patients from the screening population who were enrolled at Hubei Cancer Hospital, China, between May 2018 and November 2019. The cohort included 148 male (average age: 54.93 years [40–74 years]) and 211 female (average age: 54.91 years [40–73 years]) subjects. All the participants were eligible for sample collection and signed the written informed consent form. And all the participants were required or willing to undergo colonoscopy as prescribed by the doctor. The prospective samples were following inclusion criteria 1, 2, and 3 as applied: (1) Previous positive history of FOBT; (2) Family history of colorectal cancer; (3) Chronic intestinal disease such as diarrhea, constipation, mucinous bloody stool, appendicitis, and biliary tract disease. The clinical characteristics of the enrolled population are shown in Table 1. The novel evaluation values were significantly higher in CRC than those in Advanced adenoma (AA), non-colon cancer neoplasm (NA), healthy controls, and other cancers, which are shown in Fig. 1. Additionally, the evaluation value in AA and NA were significantly higher than those in healthy controls, while the value in other cancers were not significantly different compared to those in healthy controls (Fig. 2).

**Table 1:** The clinical characteristics of the 359 subjects who were screened

Group	N	CEA	KRAS(+)	BMP3(+)	NDRG4(+)	FOB(+)	Complex value
*Sex*							
Female	211	4.52 ± 1.06	14.69%	8.53%	22.27%	9.48%	221.7 ± 22.34
Male	148	4.53 ± 1.67	18.24%	11.49%	18.24%	15.54%	302.9 ± 33.27
*P* value		0.996	0.385	0.371	0.427	0.10	0.168
*Age*							
≥ 60y	114	6.31 ± 2.30	21.05%	14.91%	34.21%	16.67%	341.4 ± 40.02
< 60y	245	3.69 ± 0.84	13.88%	7.35%	19.18%	8.57%	207.5 ± 19.46
*P* value		0.188	0.092	0.034	0.003	0.03	0.004
*The classification*							
CRC	49	10.22 ± 4.53	46.94%	53.06%	81.63%	73.47%	1011 ± 50.38
AA	27	2.47 ± 0.23	22.22%	0	11.11%	7.41%	163.2 ± 37.94
NA	65	2.73 ± 0.20	15.38%	10.77%	18.46%	21.54%	162.3 ± 21.94
Other cancer	28	15.12 ± 8.6	14.29%	0	14.29%	3.57%	125.4 ± 23.92
Healthy Controls	190	2.38 ± 0.11	8.42%	1.58%	14.74%	1.58%	115.3 ± 8.8
*P* value		< 0.001^*^, 0.76^$^, 0.105^&^, < 0.001^#^	< 0.001^*^, 0.038^$^, 0.152^&^, 0.30^#^	< 0.001^*^, 0.999^$^, 0.033^&^, 0.999^#^	< 0.001^*^, 0.774^$^, 0.553^&^, 0.999^#^	< 0.001^*^, 0.118^$^, 0.001^&^, 0.425^#^	< 0.001^*^, 0.041^$^, 0.018^&^, 0.683^#^

^*^The comparison of CRC patients versus healthy controls

^$^The comparison of AA patients versus healthy controls

^&^The comparison of the NA patients versus healthy controls

^#^The comparison of the other cancer patients versus healthy controls

**Fig. 3:**
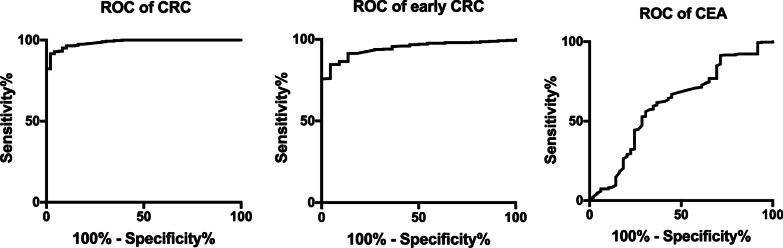
The ROC curves for the novel evaluation with sDNA tests and FIT were analyzed to assess CRC and early CRC, and the ROC curve for CEA to assess CRC. The AUC values for the novel evaluation with sDNA tests and FIT were 0.986 (0.975–0.996) for CRC and 0.945 (0.916–0.974) for early CRC. The AUC value for CEA was 0.611 (0.521–0.702)

The ROC curves of sDNA and FIT for detection of colorectal cancer are shown in Fig. 3. The sensitivity and specificity of KRAS mutation for CRC diagnosis were 46.94% and 88.39, respectively. The sensitivity and specificity BMP3 and NDRG4 of methylation for CRC diagnosis were 83.67% and 84.19%, respectively, while the sensitivity and specificity of FOB for CRC diagnosis were 73.47% and 93.55%, respectively. The sensitivity and specificity of BMP3 methylation and FOB for CRC diagnosis were 89.8% and 95.81%, while the sensitivity and specificity of NDRG4 methylation and FOB for CRC diagnosis were 93.88% and 84.84%, separately. The sensitivity and specificity of KRAS mutation and FOB for CRC diagnosis were 89.8% and 86.77%, separately. The sensitivity and specificity of the novel evaluation with KRAS mutation, BMP3 and NDRG4 methylation, and FOB for CRC diagnosis were 97.96% and 88.71%, respectively. The AUC area for the complex evaluation to diagnose CRC was 0.986 (0.975–0.996). Moreover, the sensitivity and specificity of FIT comparison for CRC diagnosis were 73.47% and 93.87%.

**Fig. 4:**
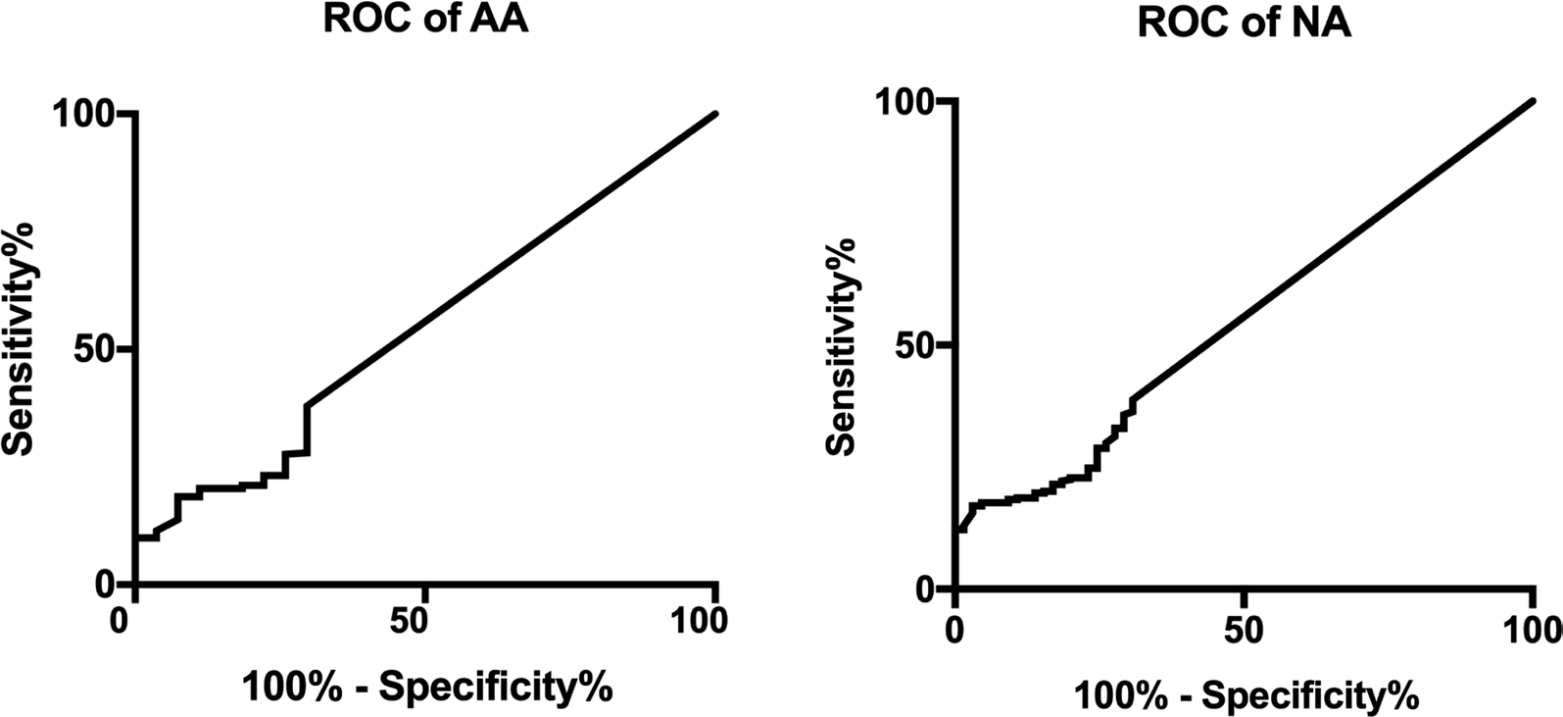
The ROC curves for the novel evaluation with sDNA tests and FIT were analyzed to assess NA and AA. The AUC values for the novel evaluation with sDNA tests and FIT were 0.543 (0.434–0.651) for AA and 0.547 (0.474–0.621) for NA

The ROC curves of sDNA and FIT for detection of advanced adenoma and non-colon cancer neoplasm (NA) are shown in Fig. 4. The sensitivity and specificity of KRAS mutation for AA and NA diagnosis were 22.22% and 73.8%, and 15.38% and 83.33%, respectively. The sensitivity and specificity of BMP3 and NDRG4 methylation for AA and NA diagnosis were 11.11% and 78.92%, and 21.54% and 74.15%, respectively. The sensitivity and specificity of FOB for AA and NA diagnosis were 7.41% and 83.73%, and 21.54% and 85.71%, respectively. The sensitivity and specificity of the novel evaluation with KRAS mutation, BMP3 and NDRG4 methylation, and FOB for AA and NA diagnosis were 29.63% and 75.69%, and 23.08% and 75.17%, respectively. The AUC area for the complex evaluation to diagnose AA and NA were 0.543 (0.434–0.651) and 0.547 (0.474–0.621), respectively.

### The correlations between sDNA and FIT and carcinoembryonic antigen (CEA) for detection of CRC

The sensitivity of CEA for detection of CRC was 30.61%, which was significantly lower than those of the novel evaluation by KRAS mutation, BMP3 and NDRG4 methylation, and FOB, while the specificity of CEA was 75.16%. The AUC of CEA was 0.611 ± 0.046 (Fig. 3).

The correlations between sDNA and FIT and clinical characteristics of populations are shown in Table 1. Of the 359 subjects in the cohort (14 patients with CRC and 345 in the screening population), 49 had CRC, 65 had NA (including 26 polyps, 19 hemorrhoids, and 20 undefined), 27 had advanced adenoma, and 28 had other cancers.

**Fig. 5:**
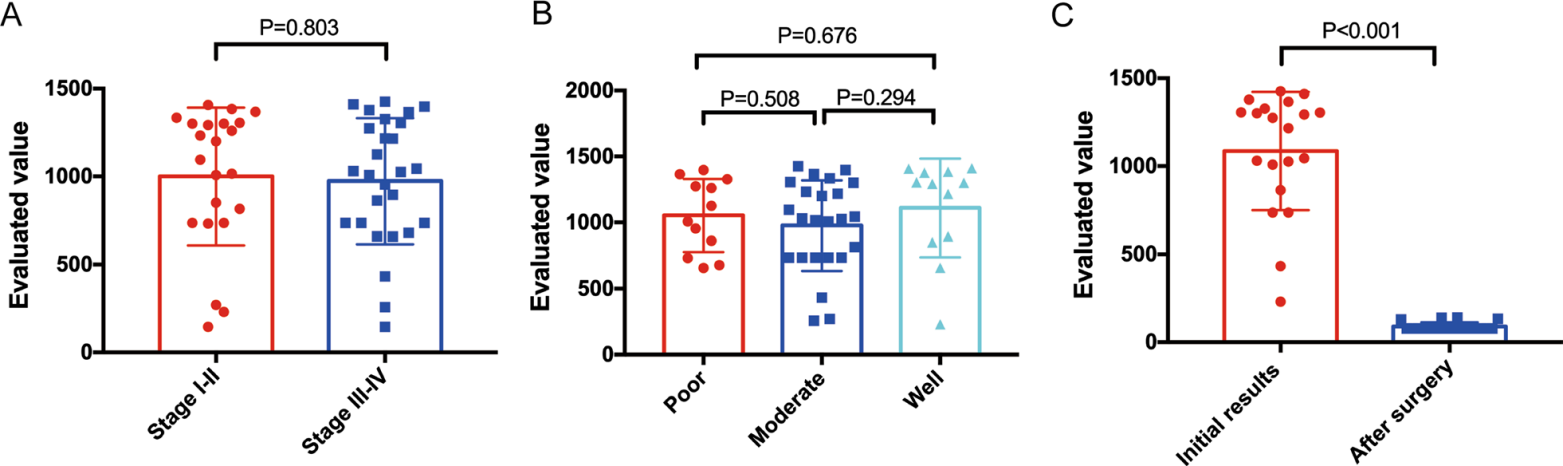
Correlation between the initial levels of evaluation value and stage, and the initial levels of evaluation value and pathology. **A** No significant differences were seen between stage I–II and III–IV. **B** No significant differences were seen among patients with well, moderate, and poorly differentiated adenocarcinoma. **C** Dynamic changes in evaluation value following surgery. The evaluation value with sDNA tests and FIT of 20 CRC patients decreased dramatically a month after resection

Of the 49 CRC patients, 22 had stage I–II disease and 27 had stage III– IV disease; further, 12, 25, and 12 had poor, moderate, and well-differentiated adenocarcinoma (Table 2). The sensitivity and specificity of the novel evaluation for stage I–II and III–IV CRC diagnosis were 95.45% and 81.6%, and 100% and 83.13%, respectively. The sensitivity and specificity of the novel evaluation for poor, moderate, and well-differentiated adenocarcinoma patients were 100% and 79.77%, 95.83% and 82.09%, and 100% and 79.83%, respectively. The levels of the novel evaluation were not significantly associated with the pathology and stage (*P* > 0.05) (Fig. 5A, B). The AUC for the complex evaluation to diagnose early CRC was 0.945 (0.916–0.974).

**Table 2:** The clinical characteristics of 49 patients with CRC

Group	N	CEA	KRAS(+)	BMP3(+)	NDRG4(+)	FOB(+)	Complex value
*Sex*							
Female	25	14.07 ± 8.03	48%	64%	80%	72%	1014 ± 70.4
Male	24	5.49 ± 2.11	45.83%	41.67%	83.33%	75%	1000 ± 76.19
*P* value		0.351	0.999	0.156	0.999	0.999	0.992
*Age*							
≥ 60y	24	12.9 ± 7.14	50%12	54.17%	83.33%	80%	1047 ± 68.52
< 60y	25	6.63 ± 4.69	44%	52%	80%	64%	960 ± 75.57
*P* value		0.001	0.999	0.999	0.999	0.345	0.675
*The clinical classification*							
Stage							
Stage I–II	22	2.94 ± 0.95	45.45%	59.09%	86.36%	86.36%	974 ± 68.91
Stage III–IV	27	16.15 ± 8.06	59.09%	48.15%	95.45%	62.96%	1001 ± 83.41
*P* value		0.003	0.547	0.568	0.488	0.104	0.803
Pathology							
Poor	12	11.97 ± 8.04	41.67%	58.33%	83.33%	66.67%	1054 ± 79.58
Moderate	25	17.18 ± 11.79	44%	44%	80%	88%	977.4 ± 69.91
Well	12	2.95 ± 3.82	58.33%	66.67%	83.33%	50%	1111 ± 107.8
*P* value		0.741^*^, 0.10^&^, 0.40^#^	0.999^*^, 0.684^&^, 0.495^#^	0.495^*^, 0.999^&^, 0.295^#^	0.999^*^, 0.999^&^, 0.999^#^,	0.183^*^, 0.68^&^, 0.04^#^	0.508^*^, 0.676^&^, 0.294^#^

^*^The comparison of the poorly affected patients versus the moderately affected patients

^&^The comparison of the poorly affected patients versus the well affected patients

^#^The comparison of the moderately affected patients versus the well affected patient

### The novel evaluation of sDNA and FIT for monitoring of CRC

The sDNA and FIT evaluation were detected after surgery in 22 patients at one month following resection. The novel evaluation exhibited a considerable decrease after resection (Fig. 5C), and the comprehensive values were below threshold (< 165) in 20 out of 22 patients. We also found residual polyps in two out of 22 patients with positive threshold value.

## Discussion

Screening for CRC is crucial as it can improve the outcome for patients diagnosed at an early stage [18]. Stool DNA (sDNA) tests and fecal immunochemical test (FIT) were evaluated for CRC screening in the clinic [19–21]. In this study, we use a novel evaluation based on the results of sDNA and FIT to diagnose CRC and monitor the treatment of CRC in Hubei Province. Our results showed that the novel evaluation with sDNA and FIT had high sensitivity and specificity for the early detection of CRC. According to the results of KRAS gene mutation, BMP3 and NDRG4 methylation, and FOBT, we combined the above detection and analyzed the results using software. Then, the comprehensive value was calculated by the software, which determined the screening results of CRC according to the cut-off value.

Colonoscopy has been considered the conventional screening method for CRC in the past decade; however, because it is not accepted by the general public, the morbidity and mortality rates of CRC are still high in China. Therefore, the noninvasive stool tests with DNA and FIT appeared to be attractive alternatives to screen people for CRC. Several sDNA screening tools such as KRAS gene mutation and BMP3 and NDRG4 methylation were previously reported as biomarkers for early CRC detection. In our study, the sensitivity and specificity of the novel evaluation for CRC diagnosis were 97.96% and 88.71%, respectively, which were higher than the sensitivity and specificity of KRAS mutation and BMP3 and NDRG4 methylation status, separately (46.94% and 88.39%, 83.67% and 84.19%). Moreover, the comprehensive value for CRC diagnosis (97.96% and 88.71%) was also more sensitive than those of the FIT comparison, which were 73.47% and 93.87%. The sensitivity of the new screening test was better than first-line FOBT (*P* < 0.001), while the specificity was lower than first-line FOBT (*P* = 0.032). The sensitivity and specificity of the novel evaluation for AA and NA detection were 29.63% and 75.9%, 23.08% and 75.17%, respectively. Furthermore, to our knowledge, the detection of other gastroenterological carcinomas such as esophageal cancer, gastric cancer, hepatocellular carcinoma, and cholangiocarcinoma by the novel evaluation with sDNA and FIT are limited. Hence, the specificity of the novel evaluation was excellent for CRC diagnosis. Compared with other serum biomarkers of CRC such as CEA, the sensitivity and specificity of the novel evaluation were higher for CRC detection (30.61% and 75.16% vs. 97.96% and 88.71%).

Commonly, stool contains a mixture of exfoliated cells from colonic mucosa and a small fraction of the neoplastic cells from tumor lesions in case of CRC patients [22]. Abnormal cells lead to the increase of KRAS gene mutation, BMP3 and NDRG4 methylation, and hemoglobin in the fecal samples. These changes were significantly correlated with the progression of colorectal neoplasms. Exfoliation of these tumor cells into stool logically occurs earlier than vascular invasion into blood. Thus, the detection of such aberrant DNA in fecal samples is ideal for specific screening for early CRC. As seen in our results, the sensitivity and specificity of the novel evaluation for early CRC detection were 95.45% and 81.6% for patients with stage I–II disease. Moreover, the sensitivity and specificity of the novel evaluation for CRC patients with stage III–IV cancer were 100% and 83.13%. We also found that the comprehensive value of such evaluation was not related with the stage and pathology of CRC, as the values were not higher in patients with advanced CRC than in those with early CRC, as well as in three types of differentiated adenocarcinoma. Interestingly, the results of the novel evaluation in 20 CRC patients became lower than threshold (< 165) after surgery. The other two patients with positive threshold value were found residual polyps. In our study, the sensitivity and specificity of the novel evaluation with KRAS mutation, BMP3 and NDRG4 methylation, and FOB for non-colon cancer neoplasm (NA) diagnosis was 23.08% and 75.17%, respectively.

## Conclusion

Previous studies have mostly investigated the detection of sDNA and FIT for CRC screening, but the comprehensive evaluation of such results by software for CRC diagnosis and monitoring has, to our knowledge, been reported for the first time in this study. It is helpful for the clinical use of sDNA and FIT. Thus, the novel evaluation may particularly be important as an indicator of early CRC and cancer progression. Additionally, the dynamic changes of the comprehensive value were correlated with the treatment and metastasis of CRC.

One of the limitations of our study is the small sample size. Future investigations on the novel evaluation with sDNA and FIT for CRC monitoring should be performed on a larger sample. Additionally, because of the limited follow-up time, the relationship between the comprehensive value and overall survival could not be explored. Therefore, an analysis with a longer follow-up duration would be highly useful.

Thus, the present findings indicate that the novel evaluation with sDNA and FIT might be a potential indicator of early CRC and a predictor of recurrence in CRC patients in Hubei Province. Thus, an efficient and reliable evaluation to measure sDNA and FIT from CRC patients could prove useful to predict the prognosis and recurrence of these cancers, and this could help clinicians select an optimal and customized management strategy for the treatment of these cancers.

## Data Availability

All data generated or analysed during this study are included in this published article.

## Abbreviations

CRC: Colorectal cancer
ROC curve: Receiver operating characteristic curve
sDNA: Stool Deoxyribo Nucleic Acid
FIT: Fecal immunochemical test
KRAS: Kirsten rat sarcoma viral oncogene
NDRG4: N-myc downstream regulated gene 4
BMP3: Bone morphogenic protein 3
PCR: Polymerase chain reaction
AA: Advanced adenoma
NA: Non-colon cancer neoplasm
OS: Overall survival
TNM: Tumor node metastasis
ARMS: Amplification-refractory mutation system
ACTB: Actin cytoplasmic 1
FOB: Fecal occult blood
CEA: Carcinoembryonic antigen
AUC: Area under curve
